# School Nurses’ Experiences With Health Dialogues: A Swedish Case

**DOI:** 10.1177/10598405211022597

**Published:** 2021-06-22

**Authors:** Catrine Kostenius

**Affiliations:** 1 Department of Health, Education and Technology, 5185 Luleå University of Technology, Sweden

**Keywords:** qualitative research, health education, health promotion, health literacy, health dialogue, school nurses, health interview

## Abstract

The aim of this study was to describe school nurses’ experiences with health dialogues and elicit their thoughts about how schools can reach the full potential in promoting students’ health literacy and learning. The phenomenological analysis resulted in four themes: (i) A golden opportunity…or not, (ii) Like a double-edged sword, (iii) Able or unable organizations, and (iv) Visions of good conditions for health and learning. School nurses’ experiences revealed that health dialogues are beneficial and can be valuable tools in promoting health and learning when (1) the health dialogues are an important part of the educational assignment, (2) school nurses are valued for fulfilling the educational assignment, and their work conditions are reasonable, (3) the results from the health dialogues and health questionnaires are used systematically to promote health and learning, (4) a “whole-school approach” is used to build enabling relationships among all school staff and students.

The educational system has a societal responsibility to create conditions for academic success that aid students’ development into prosperous and healthy citizens (Mitra et al., 2016). It is well known that student’s health impacts their learning abilities and increases their possibilities of reaching educational goals (Busch et al., 2017; Correa-Burrows et al., 2017; Dadaczynski et al., 2019). This well-established connection between health and learning increases the importance of health care professionals in schools being involved in promoting students’ health and establishing a health-promoting school environment (Hoekstra et al., 2016; Paakari & George, 2018).

Student health is promoted in various ways. School nurses worldwide are working to fulfill this responsibility in their unique roles as school-based clinical experts, care coordinators, and student advocates (Schroeder et al., 2018). Research shows that the health dialogues that students have with their school nurses can be helpful in promoting student health (Rising Holmström et al., 2013). The presence of a school nurse is associated with reduced absenteeism and missed class time (Yoder, 2020). School nurses have expressed gratitude for having a profession that impacts students’ lives (Jönsson et al., 2017). However, in recent years, school nurses have experienced challenges in the transformation of their role from mainly medical care and disease prevention to a primarily health-promoting focus (Rising Holmström et al., 2015). School nurses also express the challenges of limited time and resources, communication barriers, and multiple documentation requirements (Lineberry et al., 2018). Although it is desirable to integrate personal, social, and health education into the mandatory teaching, school nurses found that health and education are commonly treated in parallel tracks in their schools and are managed by different authorities (Rosvall & Nilsson, 2016). School nurses need good working conditions to fulfill their disease-prevention and health-promoting assignments (Rising Holmström et al., 2015). The strongest predictor of burnout among school nurses is a heavy workload and a lack of community (Jameson & Bowen, 2020).

The World Health Organization (WHO) argues that schools contribute to life-long health benefits by creating the conditions for students’ achievement through the school environment (WHO, 2018a). The WHO states that empowering young and future generations to make healthy decisions can be achieved by promoting health competencies and health literacy. The WHO’s (1986) definition of health encompasses physical, mental, and social well-being and is a resource for daily life. Health and well-being are the basis of health promotion and is described in the Ottawa Charter (WHO, 1986) as the “process of enabling people’s increased control over their own health in order to improve it…to reach a state of complete physical, mental and social well-being, an individual or group must be able to identify and realize aspirations, to meet needs, and to change or manage the environment” (p. 1). Cygan et al. (2020) remind us that although school nurses play an integral role in student health and wellness, there is a need to further understand school nurses’ impact on student health and academic outcomes through school health policy (Cygan et al., 2020).

This study was carried out in Sweden and the national and regional contexts are described in national policy documents by the Swedish National Board of Health and Welfare and Swedish National Agency for Education (2016). These agencies clarify that both school nurses and teachers have health-promoting assignments. In addition to creating good conditions for learning and student safety, school principals are responsible for the student health service staff including school physicians, school psychologists, counsellors, and school nurses (SFS, 2010:800). According to the Swedish Education Act, all students are entitled to a minimum of four health dialogues (also called health visits) during their mandatory education. The health dialogues are regulated by the Swedish Education Act and ensure that all students are offered health dialogues when attending kindergarten, 4th and 7th grades, and their 1st year of high school (SFS, 2010, p. 800). The students are invited by the school nurse to schedule a health dialogue during school hours, and the caregivers can be invited to participate (Region Norrbotten, 2020). The health dialogues are held in strict confidentiality because they are covered by Swedish Publicity and Secrecy Act (SFS, 2009, p. 400).

There are several factors to consider for the school nurses to fulfill their assignments according to the Education Act (SFS, 2010, p. 800): a good learning environment for the students to achieve their educational goals and offering all students equal student health services. The most recent recommendation made by the Swedish National Board of Health and Welfare (1998) is that 400 students per school nurse be considered a reasonable workload. However, these recommendations are not sufficient according to the Swedish School Nurses’ Association (2019) because there are a number of additional aspects to consider: (i) the number of principals for the school nurse to collaborate with, (ii) the amount of student health service meetings to attend, and (iii) number of students in primary and secondary special education, and (iv) the number of newly arrived migrant students. The Swedish School Nurses’ Association (2019) also argue that the socioeconomic composition of the student base, number of schools per school nurse, health visits, vaccinations, and follow-ups need to be considered

The health dialogue has been described as a student-centered dialogue to gain knowledge about and insight into health and lifestyle (Golsäter, 2012). The concept of health dialogues—then called health interviews—was introduced by school nurses in the student health services in Southampton, England, in the late 1980s to provide a dynamic holistic approach to identify and meet the changing health needs of students (Neylon, 1993). The health dialogue can increase students’ knowledge of health issues (Borup & Holstein, 2010) and health literacy (Paakari et al., 2019).

According to Sorensen et al. (2012), health literacy “entails people’s knowledge, motivation and competences to access, understand, appraise, and apply health information in order to make judgments and take decisions in everyday life concerning healthcare, disease prevention and health promotion to maintain or improve quality of life during the life course” (p. 3). To promote the social aspect of health literacy, an ethical and open school community in which everyone has an equal voice has been shown to be instrumental (Paakari & George, 2018). Health literacy is one of the most important skills for living a good and healthy life; making room for its practice in school is essential (Kickbusch, 2012).

One challenge with the health dialogue is the adaptation of the dialogue based on each student’s needs and wishes (Golsäter, 2012). To address this issue, students take a standard questionnaire before the health dialogue with the school nurse. According to Rising Holmström et al. (2013), the health questionnaire is a valid instrument for measuring students’ self-reported-health and identifying positive health factors. Students’ self-reported health from the health questionnaire illuminates contextual and personal assets central to students’ school experiences, health, and well-being (Forsberg et al., 2019). Although the health dialogue is helpful toward promoting students’ health, there is more work to be done to fully integrate the practice of health dialogues and health questionnaires in schools (Rising Holmström et al., 2013, 2015). Additionally, Arnesdotter et al. (2008) revealed that schools in Sweden only use a fraction of all available information from the health dialogues.

## Aim

The aim of this study was to describe school nurses’ experiences with health dialogues and elicit their thoughts about how schools can reach the full potential in promoting students’ health literacy and learning.

## Method

Inspired by van Manen (1990), a phenomenological design and a qualitative participatory approach described by Ghaye et al. (2008) was used to capture school nurses’ experiences with the health dialogue and their thoughts about how schools can reach the full potential in promoting students’ health literacy and learning. The participants were invited to share their thoughts in an open letter, which is semi-structured open-ended writing (Kostenius, 2008) to gain a deeper understanding of the nature and meaning of everyday experiences in line with van Manen (1990). The open letter was used in combination with interviews to collect the participants’ experiences and thoughts.

### Context and Participants

The northernmost region in Sweden (Norrbotten) has 14 municipalities. Seventy-one percentage of the students in that region participated in a health dialogue and filled out the health questionnaire during the 2017–2018 school year (Public Health Center, 2019). There are, however, local differences. In one municipality, only 58% of the students participated in the health dialogue and 92% in another. This particular region uses a system in which all students fill out a questionnaire before meeting the school nurse in a health dialogue. The data from all the health questionnaires are administered by the regional Public Health Center (2019). Furthermore, the center facilitates the regional health questionnaire database, compiles the results, and writes a general report to inform the 14 municipalities in the region. School nurses from all 14 municipalities in Norrbotten were invited to participate. The invitation was sent out via the regional school nurse network reaching approximately 50–55 school nurses employed at the time of the invitation. Forty-four school nurses from across the region agreed to participate. They came from urban and rural municipalities ranging in size from approximately 2,700 to 78,000 inhabitants. They all wrote open letters and 10 of them agreed to be interviewed. The interviewees had between 1 and 20 years of experience in the profession, holding health dialogues with students in kindergarten, middle, junior, and high school. No additional information was collected due to the risk of jeopardizing the participants’ confidentiality in the small municipalities.

### Ethical Considerations

Following the Helsinki Declaration (World Medical Association, 2008), oral and written information about the study were provided to the regional network for school nurses. Oral and written information about voluntary participation and confidentiality were also provided in accordance with Swedish ethics laws (SFS, 2003:460). All participants participated willingly; the study was approved by the regional ethics committee in Umeå before data collection (2017/403-31).

### Data Collection

The author visited the regional network for school nurses and met on three different occasions. All school nurses were invited to participate in the study and were given open letters on paper. To facilitate participation, the open letters were also distributed in digital form by the manager of the regional network for school nurses. Through the provision of the beginning of a sentence, the participants were encouraged to think of one or many health dialogues and describe their experiences in writing. The following sentences were used: “Now I will tell you about my experiences of holding health dialogues…” and “To use the health dialogues to their fullest potential to promote students’ health and learning, I think that.…”

van Manen (1990) argues that writing can bring individuals closer to their experiences, and this writing task gave the school nurses an opportunity to share their experiences, thoughts, and ideas. A total of 44 school nurses filled out the open letters, and 10 of the school nurses who agreed to be interviewed met the author at the office of the Norrbotten Association of Local Authorities or on the phone. The interviews started with each nurse’s unique story about their experiences with health dialogues and their thoughts about how schools can reach the full potential in promoting students’ health literacy and learning. They were prompted to widen the scope of their lived experience of holding health dialogues, for example, What happened then? What do you think about that? and Tell me more. The interviews were taped and were later transcribed verbatim by a research assistant.

### Analysis

Analysis of the data consisted of open letters and interview transcripts. This was done by the author and four colleagues in two stages: seeking meaning and theme analysis inspired by van Manen (1990). In the first step, the team set out to seek meaning by reading all the open letters and interview transcripts several times to obtain a sense of the whole. The second step was theme analysis where the team identified the experiential structures found in the data and looked for differences, similarities, and patterns. The four colleagues also engaged in discussions of the data with the author on several occasions during the analysis process.

The school nurses’ experiences with the health dialogue were organized into broader themes. The second step also included member-checking to ensure that the data were truly reflecting the school nurses’ experiences, which strengthens the trustworthiness of a study (Polit & Beck, 2004). The themes were discussed at a meeting with the regional network for school nurses guided by the following questions: Do you recognize your own individual experiences of the health dialogue in these themes? Do these themes reflect your thoughts about how schools can reach the full potential in promoting students’ health and learning? and Would you like to change or add additional experiences, thoughts or ideas? Many additions and changes were made based on the school nurses’ suggestions before the final themes were formed. Illustrative quotations from the open letters and the interview transcripts were added to exemplify the themes and give voice to the school nurses, which enhance credibility (Polit & Beck, 2004).

## Results

The phenomenological analysis resulted in four themes with two sub-themes each, revealing a large variety of experiences ranging from positive and hopeful to negative and powerless (Table 1).

**Table 1.** Four Themes, Which Encompass Two Sub-Themes Each, Illuminate School Nurses’ Experiences of the Health Dialogues and Their Thoughts About How Schools can Reach the Full Potential in Promoting Health and Learning.

Themes	Sub-themes
A golden opportunity…or not	Extending a helping hand Feeling stressed and powerless
Like a double-edged sword	Benefitting from a useful tool Being frustrated with the uncalibrated instrument
Able or unable organizations	Feeling supported and respected Being left out in the cold
Visions of good conditions for health and learning	Building an enabling school culture Creating a supportive place

### A Golden Opportunity….or Not

The school nurses described the pros and cons of the health dialogue. This is further described in the subthemes *Extending a helping hand* and *Feeling stressed and powerless*.

#### Extending a Helping Hand

According to the school nurses, health dialogues are a great resource to promote students’ health and prevent disease. In their narrations, a sense of appreciation surfaced with experiences of feeling grateful that they were in a position to extend a helping hand to individual students and their families as well as to student groups. “I follow the students from preschool class to high school and I think it is absolutely fantastic. This enables me to build a relationship with each student.” The school nurses found the opportunity to meet an individual student’s needs to be especially joyful and described the feeling of success when they could connect with students. The school nurses tailored the health dialogue to meet an individual student’s needs: “I usually hold the health dialogues while walking, which makes the students talk more.” According to the school nurses, the health dialogue lowers the threshold for students to ask for help. They also described the good feeling fostered by being a student advocate. “If there is something particular in a class…when several students have mentioned something that they are anxious about, like bullying or someone being left out and things like that, I act on it.”

#### Feeling stressed and powerless

The school nurses discussed the conditions that were out of their control making it impossible to successfully hold health dialogues, which caused them stress. The school nurses did not have enough time to spend with each student. Their heavy administrative workload limited the time available for follow-ups. “I have limited time, so I have to cut down on the (health dialogue) to be able to prepare all the paperwork, to distribute all these health information forms, the informed consent forms (to the guardians) from the county council and the municipality.” The school nurses experienced difficulties in talking undisturbed during the health dialogue, which resulted in feelings of inadequacy. “It’s about having time to talk; I am often disturbed. It does not help to place a do-not-disturb sign on the door and lock it…there is always someone knocking, knocking, jerking, jerking, knocking, knocking.”

Another challenge that the school nurses described was the large number of students. Some of the school nurses had to serve several hundred students. “Five hundred and sixty students in all grades and four units….it’s a lot.” The school nurses felt stressed when they were not able to spend enough time with students who needed support and when they did not manage to connect with the caregiver who sometimes was also in need of assistance. The nurses experienced themselves as lacking proper resources to meet the students’ needs that came up in the health dialogues, which made them feel powerless. “We are supposed to help students/families to make lifestyle changes, which is very difficult. Almost too tough!”

### Like a Double-Edged Sword

Although the health questionnaire was developed to aid the school nurses in holding the health dialogue, they described it as a double-edged sword. This is further described in the subthemes *Benefitting from a useful tool* and *Being frustrated with the uncalibrated instrument.*

#### Benefitting from a useful tool

According to the school nurses, the health questionnaire is a useful tool with many benefits. It ensures that the most important aspects of health are covered during the health dialogue. “The questions in the questionnaire are really good so it is a good approach, I think. Because all the parts are covered.…” The school nurses described the health questionnaire as a helpful point of departure for starting the health dialogue with the students regarding a wide variety of health topics such as nutrition, exercise, sleep, and screen time. Some questions are sensitive, such as those involving possible abuse and bullying. The fact that all students answer the same questions can eliminate the risk that students feel singled out. “I think that when it comes to delicate issues, it’s not so strange that I ask or want to talk about areas that can be sensitive because everyone gets the same questions.” According to the school nurses, another positive aspect of the health questionnaire is the possibility of ensuring that students are treated equally because all students answered the same questions.

The school nurses described the health questionnaire as an educational instrument and learning opportunity. “I just got an idea…the students can make statistics of their own results from the health questionnaire in math class to see what their schools’ results look like, and calculate (the health questionnaire results) on a school level to avoid being too personal.” The school nurses used the results for follow-up meetings with individual students, a group of students, and entire classroom settings. “I go through the questions with a class and talk a little about health in general and open up for a discussion or a dialogue with all the students. I do that in all classes…then they get to answer the questions individually too.” The results from the health questionnaires helped the school nurses to communicate and solve problems together with other professionals and school leaders

They stressed the importance of not making the teaching staff feel excluded: “The principal, school nurses, counsellors, and special educators are part of this local student health team, and meet regularly…it’s not us counselling them. Rather, we call it an open student health team meeting, which is a good way to organize (our work).” According to the school nurses, sharing the health questionnaire results outside of the school organization has proven to be helpful, for example, at parent meetings and at visits with local and national politicians with increased support for sustainable health-promoting efforts on the individual and organizational levels. “The principal is on the school committee, and thus, I think the results (from the health dialogue questionnaire) contributed to hiring another school counsellor last year after the increase in (students’) mental illness was presented.”

#### Being frustrated with an uncalibrated instrument

The school nurses described negative and frustrating aspects of the health questionnaire. The questions were written for older students, which meant nurses spent much time interpreting questionnaire questions for younger students who did not understand them. “I have noticed that the students in Grade 4 do not understand; thus, I have to explain many of the questions.” The extensiveness of the health questionnaire leaves no room for students to ask questions—The students rush to finish the questionnaire and often get tired. “The students ask, ‘Must I answer this?’ and it’s often at the end of the questionnaire.” The lack of time and space for the students to bring up their own thoughts about their health makes the school nurses’ work more like an assembly line than a helpful encounter. The school nurses also have doubts about how reliable the health questionnaire results are: They believe that students sometimes answer in the way that they think the adults want them to answer.

The school nurses described challenges finding the time and having necessary knowledge to compile the results. The sharing of negative results from the health questionnaire was problematic because the results were too general, and the timing of sharing the results was described as another obstacle. The school nurses were often invited to attend a meeting for caregivers arranged by the class teacher at the beginning of the term when the current results from the health questionnaire were not available. “The parent meeting is in September, and the results are ready later in October–November // When you share the questionnaire (results) from the previous school year, it may not even be their own children who have answered the questionnaires.” The school nurses also voiced frustration about the bureaucracy that prevented them from freely sharing the results in order to help the students. “The secrecy act is both good and bad, but it would be a lot easier if I could give information to the teachers…the secrecy can make my work a bit more difficult.”

### Able or Unable Organizations

The school nurses described working in able or unable organizations. This is further described in the subthemes *Feeling supported and respected* and *Being left out in the cold.*

#### Feeling supported and respected

According to the school nurses, they felt supported in school organizations that prioritized health promotion. They talked about school leaders, student health service managers, and coworkers who were interested in and willing to learn about the health dialogues. “We have decided that student health begins in the classroom. It should also be a mandatory item on the agenda in the staff meetings where one can raise issues and where one can have collegial counselling to support each other.” The school nurses described a school organization that enables cooperation and in which all staff view health and learning as a shared responsibility. “I believe that a well-functioning student health service team at school can work magic. However, to succeed, everyone in school must promote health—all staff from the school restaurant, the school janitors, the teachers, etc.” In organizations that rewarded shared learning, the health questionnaire was seen as a contribution to the educational assignment, and the school nurses were valued actors in the implementation. “The results from the health questionnaires to promote students’ health and learning must have a naturally incorporated place in the schools (organization), which is already the case in many schools.”

The school nurses shared success stories about school leaders who prioritized time to address health questions, and coworkers who were eager to gain knowledge about the health and learning connection and the results from the health questionnaires. Willingness among the school staff to put together the “knowledge puzzle” in order to help students be healthy and reach their educational goals made the school nurses feel respected. In such enabling organizations, the information that the school nurses presented was taken seriously, and health questionnaire results were in demand. All students were offered a health dialogue but some students did not attend. However, the school nurses noticed a positive difference in attendance when the teaching staff supported the health dialogue. This type of collaboration was the key to success according to the school nurses: “I believe in cooperation. The school counselors and the teachers can do so much together (with us school nurses).” The school nurses talked about parents and caregivers who respected them and were interested in discussing their children’s health and well-being: “I have been to parent meetings and I tell them a little about children’s need for (sufficient) sleep, that they need to eat breakfast and should not sit too long in front of screens and I try to support the parents…The children need good conditions (at home) so you have to have the parents onboard.”

#### Being left out in the cold

The school nurses discussed school organizations in which they were left on their own to fulfill their health-promoting and disease-prevention assignments. Health and education were treated on parallel tracks in organizations in which students’ health and learning were viewed as separate; health was more or less ignored by school leaders. “The principal doesn’t prioritize student health work.” According to the school nurses, the low status of health questions led to silos within the school organization in which they were separated from the school staff. They described “them-and-we” structures with an inability to meet and collaborate. “We need the teachers to understand the value of the health dialogues…we need to motivate the students, to build a relationship with them.” The school nurses were frustrated that they were viewed as the last resource and were called on by the teaching staff to fix health problems, learning difficulties, or social challenges in a classroom when these had become severe. “I need more knowledge and a greater mandate to work for health promotion in collaboration with other student health professionals, pedagogical staff, and the principal.”

The school nurses report that time is a scarce commodity. Without a committed school leader to put the health questions on the agenda, they were left alone with their health dialogue assignment. “There is no room to work with the teachers. Student health is put aside, and the teachers keep to themselves; there is way too little time to think together.” Continuing education was often lacking though it is essential for the success of health promotion efforts in schools. “We need continued inspiration and education on issues related to health, e.g. fundamental values, school social environment, bullying; it’s not just about the body, sleep, diet, and exercise.” In an organization in which the school nurses experienced little or no understanding from other professions, the lack of interest in the results from the health questionnaire was evident. The school nurses talked about not being invited to the parent meetings held by the teaching staff. This led to parents being informed about students’ educational outcomes by the teachers; however, the school nurses were on their own in arranging a meeting to inform parents about the results of the health dialogue questionnaires. The school nurses talked about how school staff did not have enough knowledge to work with health and learning assignments. “During this past school year, our team has talked and discussed what (disease) prevention and health promotion entail…I think many people confuse the concepts.”

### Visions of Good Conditions for Health and Learning

When looking at the future, the school nurses shared thoughts about how schools can reach the full potential in promoting students’ health literacy and learning. This is further described in the subthemes *Building an enabling school culture* and *Creating a supportive place.*

#### Building an enabling school culture

Looking at the future, the school nurses suggested aspects that could build an enabling school culture among all school staff, students, and families. They pointed out relational aspects, activities, and attitudes that could encourage the use of the health dialogues to help the schools reach the full potential in promoting students’ health literacy and learning. According to the school nurses, the school leaders’ role is crucial. The school organization must acknowledge the dual assignments for health and learning to be on the agenda simultaneously. They suggested that the school leaders include the health assignment in the systematic quality development in school. “The dream scenario, as I see it, would be that all principals include the results of the health dialogues in their quality report.”

The school nurses reported that their stress diminished, and their engagement increased when they felt secure and unafraid of being exposed in a negative way. The school nurses noted a win–win situation reaching the health and educational assignments simultaneously when school leaders developed a forum for collegial learning. The school nurses described an organization in which collaboration is the key to success and a way to counteract a negative attitude signaling (“it’s not my table”) related to the health assignment: “I (want to) become involved in the teachers’ planning to be able to work together so the teachers feel that we are there for them and do not feel that we are a threat but rather instead that we can benefit from each other’s competencies.”

The school nurses also acknowledged their own role in building trusting relationships and sharing the results from the health questionnaire with their colleagues. “I should perhaps better inform the other staff and teachers when I am holding the health dialogues” and “Well, I think I should maybe present an overall review of the (health questionnaire) results for the teachers.” According to the school nurses, having a colleague with whom they could work side by side would increase their capacity to meet students’ needs. “It would be absolutely ideal if I had a colleague on a part-time basis who could assist me when I hold the health dialogues…one could handle the students who need (acute) help…while the other one holds the health dialogues.” Some schools were well on their way to arranging for good conditions to collaborate among staff including aiding their work with health and learning assignments. “The student counsellor has been here only once a week (before) but now she’ll be here 60% so that feels great. We might even be able to start something together.”

The school nurses reported that an enabling school culture is a school organization that continuously develops to meet all students’ needs as a group and as individuals. Making room for individual arrangements should be done without putting blame or shame on the students who experience difficulties. “We try to shift focus from the individual student who does not seem to be able to make it. Perhaps it is the organization (?)…instead of lifting out the problem from the classroom and only focusing on the student, (we need to) ask ourselves, ‘How can I change my teaching or my relationship with the students?’” Student participation is described by the school nurses as a prerequisite for an enabling school culture: “It would be ideal if, when I notice that many students have similar concerns, I can meet the entire class and address the specific subject.” According to the school nurses, inviting students to analyze the results from the health questionnaire can become a learning opportunity. “I think that if they are involved and hear about (the results), then we will have a great discussion with the students. I think they will appreciate that they are allowed to participate, and they will surely realize the benefits.”

#### Creating a supportive place

According to the school nurses, well-developed physical and social aspects of the school can create a supportive place in which the health dialogues help the schools to reach the full potential in promoting students’ health literacy and learning. They spoke about a supportive place in terms of their own work conditions being reasonable as a precondition for students’ health equality. They also talked about how physical places matter, for example, school size. According to the school nurses, there is an unequal situation in large versus small schools in relation to resources. Examples include the fact that the students have access to a large student health service team in large schools, and facilities are often available for sporting activities, contemplation, or rest. In small schools, the school nurse often works alone without a student health service team and is available to the students only once a week or even less due to scarce resources. Allotting more time for each health dialogue would improve the conditions for school nurses and would help them to meet students’ health and well-being needs. “I would like to have the opportunity to allow one hour per student. Although it does not take that long for all students, I would at least like to have the time if needed.” They suggested that each school should have a full-time school nurse instead of nurses being assigned to many schools and districts. A full-time school nurse assigned to one school building would be able to open the door to the health office daily. This would increase the availability of a school nurse to students throughout the entire school day: “…then I would be a natural part of the school…we need to consider health equality for all students.” The school nurses suggested a common goal for students and school staff to contribute to a tranquil school environment in which everyone has the chance to feel comfortable during the health dialogue. They argued for functional and comfortable facilities to foster a fruitful dialogue for the success of the health dialogue. “The dream is to get a lot of time to hold the health dialogues and to be able to sit completely undisturbed.”

The school nurses indicated the need for schools to be organized such that the documented results from the health questionnaire could be used and discussed. They also emphasized the need to go beyond the physical place of the school building sharing the results with the students’ families and the community as well. They highlighted the benefits of prioritizing the use of the results systematically throughout all the municipalities in the northern region. “We also need to discuss across the region how the questions and answers (in the health questionnaire) are interpreted…statistics should be used fairly.” They further suggested aggregating the regional health questionnaire data to develop and expand school district and regional efforts for health promotion. “We need help interpreting the results and using the statistics in our health-promotion and disease-prevention work. It is important that the results from the health dialogues are taken seriously at the school district and used regularly and systematically in every school.”

## Discussion

The phenomenological analysis resulted in four themes: *A golden opportunity…or not*, *Like a double-edged sword*, *Able or unable organizations*, and *Visions of good conditions for health and learning*. These themes encompassed two subthemes each, revealing a large variety of experiences ranging from positive and hopeful to negative and powerless.

According to the findings, the school nurses viewed the health dialogues as an important part of the educational assignment. This is in line with national policy documents in which the Swedish National Board of Health and Welfare and Swedish National Agency for Education (2016) clarify that “student health is part of the education and is intended to support students’ development towards the educational goals” (p. 11). One way to ensure that student health is prioritized is the fulfillment of the law that health dialogues are to be offered to all students. The school nurses suggested that the health dialogues can be used to help the schools to reach the full potential in promoting students’ health literacy and learning when all professions align with the understanding that health is a prerequisite for learning and that it is a shared assignment. National policy documents explain that school nurses are part of the school health services and that teachers also have health-promoting responsibilities (Swedish National Board of Health and Welfare and Swedish National Agency for Education, 2016).

Similarly, Dassanayake et al. (2017) and Kearney et al. (2016) expressed a need for collaboration across professional and institutional borders to handle the complexity of shared assignments and competing perspectives. Similarly, the WHO (1997) Expert Committee on Comprehensive School Health Education and Promotion introduced the concept of health-promoting schools in 1995 and argued that schools are important means of influencing both the health and education of future generations. Their encouragement for assigning health is an important role in schools and is based on the common understanding that “to learn effectively, children need good health” (WHO, 1997, p. 2). We should also consider the possibility that the relation between students’ learning and their psychosocial well-being in school is more complex than commonly conceived.

The findings show that school staff and school leaders are more likely to support the school nurses in offering and administrating health dialogues when they have a basic understanding of the relation between health and learning. However, the school nurses described an ambivalent situation complicating the enactment of the health-promoting assignment in school. According to Braun et al. (2011), the issue of shared or nonshared responsibilities and a mission’s high or low priority and legitimacy in organizations is often ambiguous. Höög (2014) argues that the connection between student health work and the school’s educational assignments is often weak and that one of these perspectives is often dominant. This in collaboration can either provide a fruitful perspective or create tension and conflict.

The school nurses emphasized that health and learning are connected. They also argued that the school nurses’ assignment to offer health dialogues to all students strengthens students’ health literacy and improves students’ chances of reaching their educational goals. They experienced the value of school leaders and colleagues viewing health and learning as a shared assignment among all school staff and viewing health as a vital part of the educational assignment. Although schools acknowledge the strong connection between health and learning, there is a need for a team support for the work (Moynihan et al., 2016). The participating school nurses expressed the need for support from their school leaders. This finding echoed Garmy et al. (2015), who emphasized the importance of school nurses having full support from the school administration when implementing universal school-based health-promoting programs.

According to the findings, the health dialogues helped build valuable relationships with the students, lowering the threshold for students to ask for help. Similarly, Borup (2002) found that health dialogues helped the school nurses create a communicative room with the students—this space was the foundation for students to make changes that promote their own health. School nurses are in a position to positively influence schoolchildren’s health individually, in groups, and in the classroom (Risisng Holmström et al., 2013).

The findings show that the health questionnaires resource for strengthening health equality among students and for guiding systematic school quality development. The school nurses discussed how the results from the health questionnaire were more likely to be communicated with all staff when the school leaders prioritized taking time to address health questions. They also pointed out several faults of the health questionnaire. For example, they found it too comprehensive and, thus, taking too much time for the students to complete. Some of the questions were not suitable for younger students.

Empowerment is a central aspect of the health dialogues (Borup, 2002). Unfortunately, according to the findings, the amount of time the health questionnaire required did not leave enough time to address the students’ own agendas, decreasing the empowering aspects of the health dialogues. The lack of time could result in a one-way communication instead of a motivational dialogue with a reciprocal nature. Rising Holmström et al. (2013) noted need for an awareness of the imbalance of power in which the school nurse is an adult and must cautiously balance the interaction to avoid intimidating the students.

The findings suggest that health dialogues can help schools to reach the full potential in promoting students’ health literacy and learning when the conditions for school nurses to fulfill their assignment of promoting health in general and health equality in particular are reasonable. School nurses report that reasonable conditions depend on several aspects. Enough time must be available to conduct the health dialogues, understand the students’ perspective, and meet the students’ needs. The documentation of the health questionnaires to make use of the results to promote health and learning presupposes both time and competence. According to some of the school nurses, the number of students assigned to their workload was not reasonable. Some school nurses served several hundred students, which they found to be unreasonable. Managing multiple schools on a weekly basis made the situation untenable. This result is ominous as Jameson and Bowen (2020) found that the workload is significant in predicting school nurse burnout.

However, some of the school nurses described opposite experiences. They shared positive examples of work conditions and visions for promoting health equality among students. School leaders take responsibility for gathering all school staff, regardless of profession, and thus enabling collaboration. When the school nurses received a mandate to share the results from the health questionnaire, they acquired the necessary support to fulfill their health-promoting and disease-prevention assignments. School leaders who acknowledged competing perspectives—individual, group, and organizational—actively worked to fulfill the school’s health policy responsibility and made health equality among all students possible. According to Säljö and Hjörne (2013), there are ways of working to overcome the risk of conceptualizing social problems in school and societal challenges as individual students’ problems, for example, adding processes to improve the overall instructional process for all students in a school class.

Regarding Antonovsky’s theory on salutogenesis, Bruun Jensen et al. (2017) argue that health-promoting schools should focus on all people in the system not only on people at risk. The system should address and promote “salutary” factors and not only remove risks. According to Nilholm and Ahlm (2010), when students experienced a sense of community, their participation increased, and they had stronger feelings of security when teachers made a conscious effort to work to achieve an ideal of inclusion for all students “to create a learning community where differences are valued” (p. 239).

Based on the findings, the health dialogues can help schools to reach the full potential in promoting students’ health literacy and learning when a whole-school approach is used to build enabling relationships among all school staff and students. This is in accordance with earlier research (Adamowitsch et al., 2017; Kostenius & Nyström, 2020). Warne et al. (2017) also argue for a whole-school approach in which students’ sense of belonging is strengthened. Wong et al. (2018) noted that a school climate that supports social and emotional well-being encourages students to have health-promoting connections to their teachers. Those connections support students’ educational ambitions and plans for the future. However, schools tend to retain a traditional topic-based approach instead of realizing an integrated whole-school approach, underscoring the need for more support during implementation and cultural adaptations for health-promotion activities (McIsaac et al., 2017). The school nurses advocated sharing health questionnaire results with students, their parents, all school staff, and the outside community to strengthen the health and learning connection. Successful health-promoting schools within a national educational system require political will, leadership support from school managers, training of teachers, and partnership and mutual understanding between the education and health sectors to build trust and capacity (Young et al., 2013).

### Implications for School Nursing and School Health Services

Offering students health dialogues is not necessarily a universal school nursing practice; however, the Swedish school nurses’ experiences shared in this article may inform practice and ensure equal opportunity for all students to achieve health and educational outcomes as argued by the WHO (2018a, 2018b). Based on these findings, the health dialogue may help schools to reach the full potential in promoting students’ health literacy and learning. The focus is on participation, building relationships, and receiving adequate support. Participatory practices can build enabling relationships that empower people by giving them a voice and space in a democratic spirit (Ghaye et al., 2008). Bragg (2007) argues that when one improves schools, it is not about the school systems but rather about building relationships.

In summary, school nurses’ experiences revealed that health dialogues are beneficial and can be valuable tools in promoting health and learning when:

1. The health dialogues are an important part of the educational assignment.
2. School nurses are valued for fulfilling the educational assignment, and their work conditions are reasonable.
3. The results from the health dialogues and health questionnaires are used systematically to promote health and learning.
4. A “whole-school approach” is used to build enabling relationships among all school staff and students.

### Limitations and Recommendations for Future Research

One limitation of this study was that although the participating nurses came from a large geographical area encompassing 14 municipalities, they also came from only one out of 21 regions in Sweden. Another limitation was that the participants represented only one profession: school nurses. A more complete picture could have emerged if several different professions were included along with students. However, it is important to consider that the knowledge gained in this study about the school nurses’ assignment holding health dialogues is of value in itself. It can inform school nursing and school health services.

Ministry of Education in Sweden (2021) published a committee directive on better opportunities for students in compulsory schools including knowledge requirements, students’ equal access to school health services, and a reasonable number of school health staff per student. Future research will address the role of school nurses in overcoming sustainability challenges when promoting health literacy and learning as a shared assignment with school leaders, teachers, and other professions in the school.
